# Inhibition of Acetylcholinesterase and Butyrylcholinesterase by a Plant Secondary Metabolite Boldine

**DOI:** 10.1155/2018/9634349

**Published:** 2018-04-05

**Authors:** Adam Kostelnik, Miroslav Pohanka

**Affiliations:** ^1^Faculty of Chemical Technology, University of Pardubice, 532 10 Pardubice, Czech Republic; ^2^Faculty of Military Health Sciences, University of Defence, 500 10 Hradec Kralove, Czech Republic

## Abstract

Acetylcholinesterase (AChE) and butyrylcholinesterase (BChE) are two enzymes sensitive to various chemical compounds having ability to bind to crucial parts of these enzymes. Boldine is a natural alkaloid and it was mentioned in some older works that it can inhibit some kinds of AChE. We reinvestigated this effect on AChE and also on BChE using acetyl (butyryl) thiocholine and Ellman's reagents as standard substances for spectrophotometric assay. We found out IC_50_ of AChE equal to 372 *μ*mol/l and a similar level to BChE, 321 *μ*mol/l. We conclude our experiment by a finding that boldine is cholinesterase inhibitor; however we report significantly weaker inhibition than that suggested in literature. Likewise, we tried to investigate the mechanism of inhibition and completed it with in silico study. Potential toxic effect on cholinesterases in real conditions is also discussed.

## 1. Introduction

Cholinesterases are enzymes splitting esters of choline. We distinguish two enzymes belonging to the group of cholinesterases, AChE which is presented in nervous system and terminates neurotransmission and BChE occurring in serum but the particular function of the enzyme remains undiscovered [1, 2]. They are sensitive to broad spectrum of molecules; many of them have artificial origin (e.g., organophosphate and carbamate pesticides or nerve agents) [3]. Research on natural inhibitors which brought substances like physostigmine or galantamine was started long time ago, lately their synthetic versions were also introduced [4, 5].

**Figure 1:**
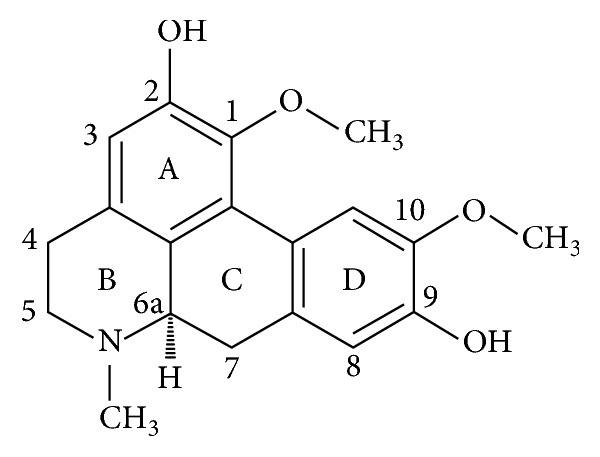
Structure of boldine.

Boldine, in chemical terminology (S)-2,9-dihydroxy-1,10-dimethoxy-aporphine (Figure 1), is a natural alkaloid with reported antioxidant activity. It was isolated from dozens of plant species from* Monimiaceae and Magnoliaceae* [6, 7] and recently it was reported to inhibit AChE with IC_50_ equal to 8.5 *μ*mol/l [8]; thus it could be potential cholinesterase inhibitor. In this work, we reinvestigated its effect on AChE and also BChE and calculated IC_50_ for both of them. We tried to propose mechanism of interaction of boldine with both cholinesterases as no evidence in literature about this interaction was found. Discussion of potential toxic effect in real conditions is also given. We hypothesize that boldine would serve as a structure for pharmacology research and, furthermore, boldine would act as a multitarget toxin. Unfortunately, data about boldine impact on targeted receptor site are scattered. We decided to investigate interaction between boldine and AChE in higher details than currently available.

## 2. Material and Methods

### 2.1. Chemicals

Acetylcholinesterase human, recombinant (expressed in HEK 293 cells, lyophilized powder, ≥1000 U/mg protein; activity for acetylthiocholine chloride was set to 64 U), butyrylcholinesterase from human serum (lyophilized powder, ≥50 U/mg protein; activity for butyrylthiocholine was set to 14 U), boldine (CAS 476-70-0), acetylthiocholine chloride (ATChCl), butyrylthiocholine iodide (BTChI) 5,5′-dithiobis(2-nitrobenzoic) acid (DTNB), and phosphate buffer saline (PBS) pH 7.4 were supplied by Sigma-Aldrich (St. Louise, MO, USA); ethanol was purchased from Penta (Prague, Czech Republic).

### 2.2. Enzyme Activity Assay

AChE activity was assayed, as triplicate, in standard spectrophotometric cuvette in following way: 400 *μ*l DTNB (1 mmol/l), 25 *μ*l AChE, 450 *μ*l PBS, and 25 *μ*l boldine ethanolic solution, and reaction was started by 100 *μ*l of ATChCl (10 mmol/l). Absorbance was measured immediately and then after 2 min of reaction. BChE activity assay was performed as follows: 400 *μ*l DTNB, 25 *μ*l BChE, 450 *μ*l PBS, and 25 *μ*l boldine ethanolic solution, and reaction was initiated by 100 *μ*l of BTChI (10 mmol/l). Difference in absorbance was measured after 2 minutes.

### 2.3. Data Processing

Enzyme activity was calculated from absorbance using extinction coefficient for 5-thio-2-nitrobenzoic acid *ε* = 14,150 l × mol^−1^  × cm^−1^ [9]. Dixon plot was constructed in Origin software (Origin, Northampton, MA, USA) and *K*_*i*_ was then calculated form Michaelis Menten equations.

### 2.4. Docking Study

UCSF Chimera (version 1.11.2; developed by RBVI with support of NIH, University of California, San Francisco [10]) was used for creating images and molecular modeling. Human AChE (PDB code: 3LII [11]) and BChE (PDB code: 1P0I [12]) were fetched and prepared for docking using Dock Prep tool (added hydrogens, assigned charges). For docking, AutoDock Vina tool was then used, and calculation was run online on Opal server.

## 3. Results and Discussion

**Figure 2:**
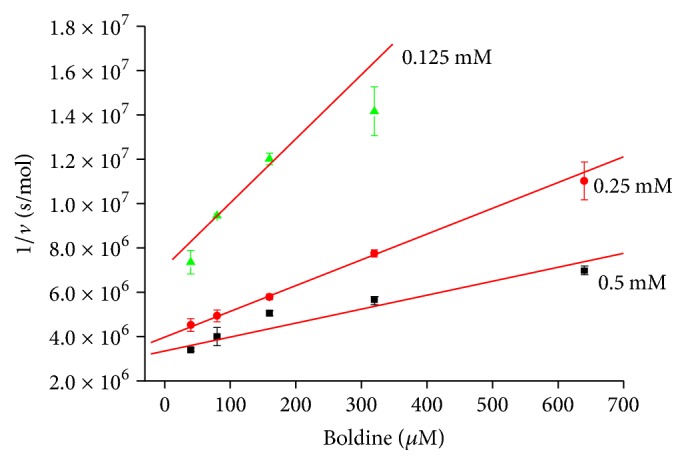
Dixon plot for AChE and different concentration of substrate (indicated beside each line). Error bars are for *n* = 3 ± standard deviation.

**Figure 3:**
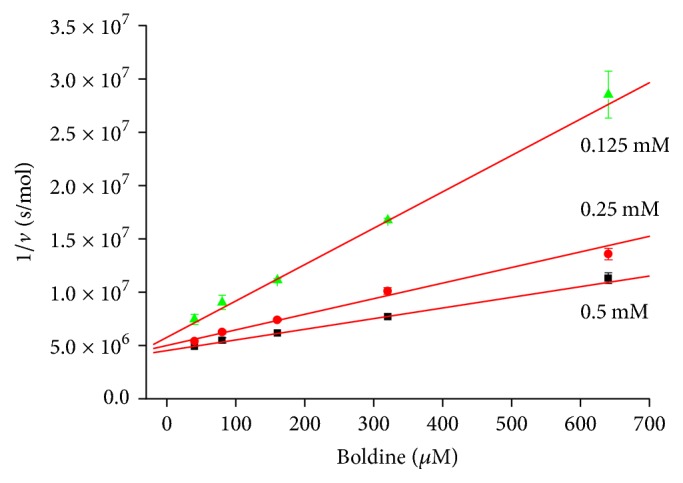
Dixon plot for BChE and different concentration of substrate (indicated beside each line). Error bars are for *n* = 3 ± standard deviation.

We investigated effect of boldine on both cholinesterases and found out that it is able to inhibit them. Dixon plot revealed noncompetitive mechanism of inhibition which is the most common inhibition mechanism for cholinesterases [13]. The inhibition of AChE by boldine was already investigated and authors reported IC_50_ 8.5 *μ*mol/l [8]. We found out *K*_*i*_ for boldine and human AChE equal to 372.30 *μ*mol/l and *K*_*i*_ for boldine and human BChE equal to 321.85 *μ*mol/l. *K*_*i*_ obtained from Dixon plot is directly proportional to IC_50_ (Figures 2 and 3). As seen in figures, our findings are not in full agreement with previously achieved result because we found out approximately 40-fold higher IC_50_ values for AChE. This difference might be seen in used enzymes, as we used pure form of human AChE, while in the quoted paper AChE is not specified, and authors refer to original paper published by Ellman, who used bovine erythrocyte cholinesterase [14]. Another difference could be in used boldine isomer where hydrogen atom in position 6a gives two optical forms. We used biologically active (S) isomer while the cited paper does not contain specification of the used isomer. Despite the fact that two isomers exist, our docking results suggest that mentioned hydrogen atom is not crucial for molecule interaction with the enzyme. From the aforementioned facts, we claim that boldine is very weak inhibitor with limited impact for pharmacological research. From the achieved results, we can also learn that IC_50_ for both cholinesterases is practically the same. This is suggesting that inhibition mechanism of boldine towards cholinesterases lies in inhibition of anionic subsite, as peripheral anionic subsite in BChE is missing [1]; thus inhibition via this site seems to be improbable. Data from experiment with both cholinesterases are summarized in Tables 1 and 2.

**Figure 4:**
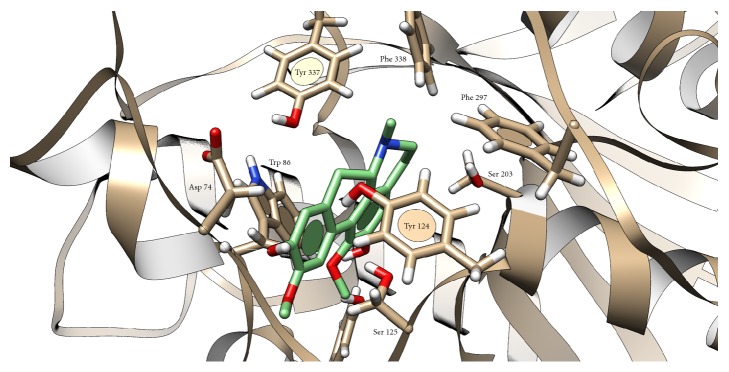
Presumed position of boldine in AChE cavity. Atom color: white = H, red = O, blue = N, grey (in AChE) and green (in boldine) = C. White line represents hydrogen bond.

**Figure 5:**
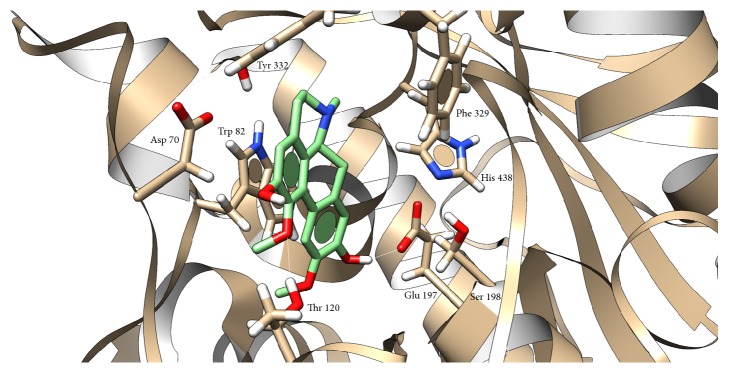
Presumed position of boldine in BChE cavity. Atom color: white = H, red = O, blue = N, grey (in AChE) and green (in boldine) = C. White lines represent hydrogen bonds.

**Figure 6:**
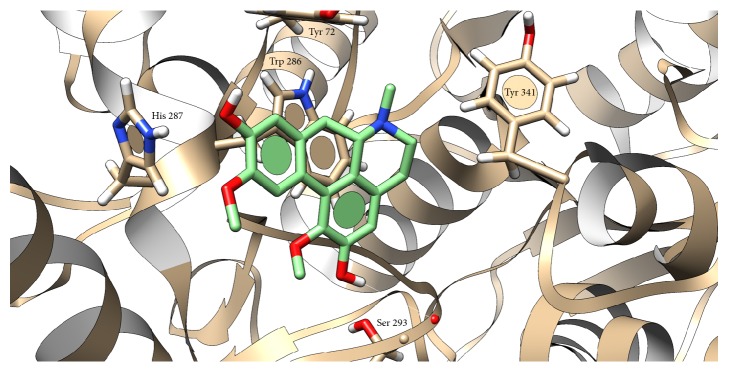
Presumed position of boldine in peripheral anionic subsite of AChE. Atom color: white = H, red = O, blue = N, grey (in AChE) and green (in boldine) = C. White line represents hydrogen bond.

Docking of boldine to cholinesterases was not found in the literature; thus we tried to investigate mechanism of this interaction. Grid box for boldine docking into AChE was set around Trp 86 and in BChE structure around Trp 82. Docking showed interaction of boldine with AChE in anionic subsite, where H-bond (2.050 Å) with Asp 74 and OH group (position 9) was predicted and stabilized via *π*-*π* interaction of Trp 86 and A ring (Figure 4). Similar situation occurred in BChE, where H-bonds between OH group (position 9) and Glu 197 (1.958 Å) and between OCH_3_ group (position 1) and Thr 120 (2.387 Å) were predicted. Stabilization is provided by *π*-*π* interaction of Trp 82 and D ring (Figure 5). Our results and also previously published docking study of another aporphine derivates [15] support the aforementioned idea about inhibition via anionic subsite of the enzymes. Another clue lies in mechanism, as noncompetitive inhibitors bind to anionic subsite of the enzyme which can be seen from examples of other cholinesterases inhibitors like tacrine and donepezil [13, 16, 17]. Some aporphine derivatives were reported to exert dual activity towards AChE, when, beside anionic subsite, interacting also with peripheral anionic subsite [18, 19]. Therefore, we checked this possible interaction and found out H-bond (2.127 Å) between OH group (position 2) and Ser 293, stabilized by *π*-*π* interaction with Trp 286 and ring A (Figure 6). Facts about boldine interaction with cholinesterases are summarized in Table 3.

Boldine is contained in various plants, widely used in Latin America for decades countries and also exported into Europe (e.g.,* Peumus boldus*) [20] and it is known as a low toxic secondary metabolite. This is supported by LD_50_ values, as 500–1000 mg/kg (p.o., mice and guinea pigs, resp.) is necessary to cause half probability of fatal poisoning [6]. Considering the fact that plasma concentration after administration of 10 mg/kg (p.o., rats) reaches approximately 1 *μ*mol/l of boldine in its peak [21], the aforementioned lethal dose 500 mg/kg would hypothetically reach 50 *μ*mol/l concentration in the blood or blood plasma. However, higher concentration is needed for significant decay of AChE activity. Thus, even lethal dose is not enough to cause cholinesterase inhibition.

## 4. Conclusion

Effect of boldine on AChE was reinvestigated, and IC_50_ equal to 372.30 *μ*mol/l was found out compared to previously published 8.5 *μ*mol/l. Thus, we claim that boldine is a cholinesterase inhibitor but it has very low affinity with the enzyme and its inhibitory effect reaches very low scale. We also investigated the effect on BChE (IC_50_ 321.85 *μ*mol/l) and it was found to be at the same level as that on AChE. We investigated inhibition mechanism of boldine to cholinesterases and proposed inhibition mechanism of boldine to AChE by docking. Results are supported by previously published findings and our experimental data and indicate inhibition via anionic subsite. We also discussed theoretical effect of boldine on AChE in real conditions. Considering the conclusions, boldine appears weak inhibitor of cholinesterases, not well suited for pharmacological research. On the other hand, its ability to inhibit cholinesterases is attributed to its biological effect though the only low manifestation is expected.

## Figures and Tables

**Table 1:** Data from AChE inhibition assay.

Substrate (mM)	Slope (s × l/mol^2^)	Interception (s/mol)	Correlation coefficient
0.5	3.34 × 10^6^	6.31 × 10^3^	0.9532
0.25	3.97 × 10^6^	11.64 × 10^3^	0.9985
0.125	7.13 × 10^6^	28.95 × 10^3^	0.9662

**Table 2:** Data from BChE inhibition assay.

Substrate (mM)	Slope (s × l/mol^2^)	Interception (s/mol)	Correlation coefficient
0.5	4.54 × 10^6^	9.99 × 10^3^	0.9983
0.25	5.01 × 10^6^	14.67 × 10^3^	0.9924
0.125	5.79 × 10^6^	34.14 × 10^3^	0.9988

**Table 3:** Found facts about boldine and cholinesterases.

Enzyme	Inhibition mechanism	IC_50_ (*μ*mol/l) by spectrophotometry	Δ*G* (kcal/mol) in silico	Anionic subsite interaction in silico	Peripheral anionic subsite interaction in silico
AChE	Noncompetitive	372.30	−7.6 (a. site) −6.2 (p. site)	H-bond: Asp 74 *π*-*π*: Trp 86	H-bond: Ser 293 *π*-*π*: Trp 286
BChE		321.85	−9.7	H-bond: Thr 120, Glu 197 *π*-*π*: Trp 82	—
